# Preclinical Neuropathic Pain Assessment; the Importance of Translatability and Bidirectional Research

**DOI:** 10.3389/fphar.2020.614990

**Published:** 2021-02-08

**Authors:** Amy S. Fisher, Michael T. Lanigan, Neil Upton, Lisa A. Lione

**Affiliations:** ^1^ Transpharmation Ltd., The London Bioscience Innovation Centre, London, United Kingdom; ^2^ School of Life and Medical Sciences, University of Hertfordshire, Hatfield, United Kingdom

**Keywords:** neuropathic pain, electroencephalography, translatability, preclinical, clinical, burrowing, endpoints, quantitative sensory testing

## Abstract

For patients suffering with chronic neuropathic pain the need for suitable novel therapies is imperative. Over recent years a contributing factor for the lack of development of new analgesics for neuropathic pain has been the mismatch of primary neuropathic pain assessment endpoints in preclinical vs. clinical trials. Despite continuous forward translation failures across diverse mechanisms, reflexive quantitative sensory testing remains the primary assessment endpoint for neuropathic pain and analgesia in animals. Restricting preclinical evaluation of pain and analgesia to exclusively reflexive outcomes is over simplified and can be argued not clinically relevant due to the continued lack of forward translation and failures in the clinic. The key to developing new analgesic treatments for neuropathic pain therefore lies in the development of clinically relevant endpoints that can translate preclinical animal results to human clinical trials. In this review we discuss this mismatch of primary neuropathic pain assessment endpoints, together with clinical and preclinical evidence that supports how bidirectional research is helping to validate new clinically relevant neuropathic pain assessment endpoints. Ethological behavioral endpoints such as burrowing and facial grimacing and objective measures such as electroencephalography provide improved translatability potential together with currently used quantitative sensory testing endpoints. By tailoring objective and subjective measures of neuropathic pain the translatability of new medicines for patients suffering with neuropathic pain will hopefully be improved.

## Introduction

Neuropathic pain arises from lesions or diseases affecting the somatosensory component of the nervous system at any level of the peripheral or central nervous system (Jensen et al., 2011). Neuropathic pain is a distinct clinical description based on common neurologic signs and symptoms despite a large variety of etiologies (Baron et al., 2010). Sleep disturbances, anxiety and depression are frequent and severe in patients with neuropathic pain, whilst quality of life (QoL) is more impaired in patients with chronic neuropathic pain than in those with chronic non-neuropathic pain that does not come from damaged or irritated nerves (Colloca et al., 2017). Reducing QoL poses a huge economic burden to the health system and society (Feldman et al., 2017).

Neuropathic pain is mechanistically heterogenous encompassing degrees of neurogenic sensitization, deafferentation and/or neurogenic inflammation. Signs and the symptoms of neuropathic pain include allodynia, hyperalgesia and paresthesia. One mechanism can underlie many different symptoms, the same symptom in two patients may be caused by different mechanisms, more than one mechanism can operate in a single patient, and these mechanisms may change with time (Woolf and Mannion, 1999). Many mechanisms are also still to be elucidated. Notably, without biomarkers that predict neuropathic pain or identification of the underlying mechanism(s), the optimum treatment strategy for the patient’s neuropathic pain cannot easily be selected or identified preclinically for successful translation.

Severity of neuropathic pain is a primary predictor of the negative health impact on patients (Doth et al., 2010). Hence the goals of therapy include improvement in pain control, coping skills and restoration of functional status. Clinically meaningful chronic neuropathic pain relief is measured in randomised clinical trials (RCTs) as a significant reduction in reported pain intensity numerical rating score (NRS) encompassing spontaneous and stimulus evoked pain (Table 1) (Finnerup et al., 2015). Pharmacological treatment represents the main option for managing chronic neuropathic pain with moderate efficacy based upon number needed to treat (Colloca et al., 2017). The anticonvulsant drug, pregabalin (Lyrica®) is the most extensively studied drug by far, with clinical studies evaluating almost 12,000 participants across eight different neuropathic pain conditions (Derry et al., 2019). Pregabalin at daily oral doses of 300–600 mg can provide at least 50% pain intensity reduction in around 3 to 4 out of 10 people compared with 1–2 out of 10 for placebo in postherpetic neuralgia and painful diabetic neuropathy patients. Given that half of those treated with pregabalin will not achieve worthwhile pain relief indicates there is significant scope for improvement (Derry et al., 2019).

**TABLE 1:** Summary of clinical and preclinical primary efficacy assessment endpoints for translation of current licenced medicines for painful diabetic peripheral polyneuropathy.

Licenced/recommended medicine year of approval in Europe daily dose (mg)	RCT Primary and secondary endpoints	Preclinical neuropathic pain model	Preclinical efficacy MED	Primary stimulus evoked sensory endpoints	Translation
^a^Pregabalin Lyrica®, (2004) 300–600 mg/day	↓ Pain intensity/quality (weekly SF-MPQ VAS score) ↓ sleep interference score Freynhagen et al. (2005), Rosenstock et al. (2005), Richter et al. (2005)	STZ (50 mg kg ip rat)	3 mg kg po 10 mg kg po	↓VFH (static allodynia) ↓VFH (Dynamic allodynia) Field et al. (1999)	^a^Forward
Gabapentin Neurontin®, (2002) 900–3,600 mg/day	↓ Pain intensity (weekly 11-point Likert score) ↓QoL, ↓sleep interference score Backonja (1998), Morello et al. (1999), Simpson et al. (2001)	STZ (50 mg kg ip rat) CCI (rat) SNL L5/6 (rat)	10 mg kg po, 10 μg i.t. 30 mg kg po, 1 μg i.t. 30 mg kg po, 100 mg kg ip 100 mg kg ip	↓VFH (static allodynia) ↓VFH (Dynamic allodynia) ↓VFH (static allodynia) ↓PWL (cold water allodynia) ↓VFH (static allodynia) Hunter et al. (1997), Field et al. (1999), Field et al., (2002)	Back/Forward (Bidirectional)
Amitripyline 25–100 mg/day (recommended)	↓Pain intensity (weekly 11-point Likert score, VAS) ↓sleep interference score/daily activities Max et al. (1992), Biesbrock et al. (1995), Morello et al. (1999)	STZ (50 mg kg ip rat) CCI (rat) SNL L5/6 (rat)	0.5 mg kg po 1.5 mg kg sc 10 mg kg ip, 60 μg i.t., 100 nmol ipl	↓VFH (static allodynia) No effect (Dynamic allodynia) ↓tonic pain score ↓PWL (Thermal hyperalgesia) No effect (static allodynia) Ardid and Guilbaud (1992), Field et al. (1999), Esser and Sawynok (1999)	Back/Forward (Bidirectional)
Duloxetine Cymbalta® (2005) 60–120 mg/day	↓Pain intensity (weekly 11-point Likert score) ↓QoL No change in dynamic allodynia Wernicke et al. (2006), Goldstein et al. (2005), Raskin et al. (2006)	STZ (50 mg kg ip rat) STZ (200 mg kg ip mouse) SNL L5/6 (rat)	20 mg kg po 20 μg i.t. 20 mg kg po 20 mg kg po	↓VFH (static allodynia), ND (Dynamic.allodynia) ↓PWL (Thermal hyperalgesia) ↓VFH (static allodynia) Iyengar et al. (2004), Mixcoatl-Zecuatl and Jolivalt (2011), Kuhad (2009)	Back/Forward (Bidirectional)

^a^ Recommended first-line treatment, RCT (randomised clinical trial), MED (minimum effective dose), STZ (streptozocin), CCI (chronic constriction injury), SNL (spinal nerve ligation), VFH (Von Frey hair), PWL (paw withdrawal latency), ND (not determined), i.t. (intrathecal), po (oral), ip (intraperitoneal), visual analogue scale (VAS), ipl (intraplantar) of the Short-Form McGill Pain Questionnaire (SF-MPQ), QoL (measures of quality of life (Short Form–36 Quality of Life Questionnaire and Profile of Mood States)), Equivalent therapeutic human doses are not determined in preclinical studies as drug plasma concentrations are not reported.

Current systemic and topical pharmacological treatments of neuropathic pain have substantial limitations in terms of the level of efficacy provided and/or the side effect profile. This means the management of neuropathic pain is unsatisfactory in both preventing its development and in halting or modifying its progression (Finnerup et al., 2015). A key to developing much needed new treatments to better manage neuropathic pain is to understand the pharmacology of novel molecules to aid their translation from preclinical species to efficacy and safety in patients. Given that there has been no translation of a new medicine for neuropathic pain since Qutenza (capsaicin 8% patch) in 2009 (Baranidharan et al., 2013) one consideration is to look more closely at the way in which neuropathic pain is modeled and measured preclinically compared to human studies (Percie du Sert and Rice, 2014; Mogil, 2019a).

The primary focus of this review is to examine the industry standard measures of neuropathic pain and analgesia in animal models and patients in combination with how bidirectional research is implementing ways to measure spontaneous ongoing chronic pain and associated QoL in animals to further improve forward translation. As will be discussed, currently industry standard markers of neuropathic pain in humans and animals are distinct and subjective. In humans this is not a problem as pain is an individual experience and subjective markers such as questionnaires accurately measure a human’s pain level, although can lead to significant variability. However industry standard stimulus evoked pain markers in animals, such as static allodynia (von Frey) are often a poor representation of the animal’s spontaneous neuropathic pain and rely on human interpretation. Translatability between these distinct and subjective industry standard markers is poor as shown by the lack of current translational success. An improvement would be to compliment simple stimulus evoked markers with an objective marker in animals such as burrowing which removes human subjectivity, but cannot be directly translated to a marker in humans. An ideal marker for neuropathic pain (and chronic pain per se) is likely one that is objective in both humans and animals and can be directly translated between the two, such as data from Electroencephalography (EEG) recordings, which can be conducted in both species with no human subjectivity involved.

## Bi-Directional Translation of Current Treatments for Neuropathic Pain

Most neuropathic pain conditions are clinically managed with a choice of four licenced or recommended medicines, amitriptyline, duloxetine, gabapentin, or pregabalin whilst carbamazepine is specifically licenced for trigeminal neuralgia (National Institute for Health and Care Excellence, 2013). These medicines (except pregabalin) were originally licenced for other therapeutic indications (gabapentin for epilepsy, duloxetine and amitriptyline for depression) and successfully back translated (this term is used interchangeably with reverse translated) from anecdotal clinical use in RCTs in neuropathic pain patients followed by preclinical efficacy in animal models.

Painful peripheral diabetic neuropathy is a widely studied condition in RCTs to assess neuropathic pain therapies as it is a leading cause of chronic peripheral neuropathic pain affecting between 25 and 50% of patients (Abbott et al., 2011; van Hecke et al., 2014). To date, there are 39 phase 2/3 RCTs in the United States/United Kingdom in patients with painful peripheral diabetic neuropathy filed on clinicaltrials.org which represents 24% of the total number of RCTs for investigating peripheral neuropathic pain. Pre-diabetes and diabetes now affect 316 million and 387 million people worldwide, respectively, and it is estimated that at least 60–70% will develop associated neuropathy complications, with prevalence increasing with duration of diabetes (Feldman et al., 2017). Furthermore, there is a positive correlation between diabetic neuropathy severity, poor glycaemic control with risk and intensity of neuropathic pain (Themistocleous et al., 2016).

We have summarized in Table 1 pivotal clinical (RCTs) and preclinical studies supporting the approval of these four licenced medicines for painful diabetic peripheral neuropathy, highlighting successful bidirectional translation of duloxetine, amitriptyline and gabapentin and forward translation of pregabalin. Duloxetine is a selective norepinephrine and serotonin reuptake inhibitor that in 2004 was the first medicine approved for painful diabetic neuropathy in the United States. This was based upon reducing spontaneous pain intensity at 60 mg once or twice per day and reversal of nociception and static mechanical allodynia in rodent face validity models of peripheral neuropathic pain. A few RCTs reported significant improvement in spontaneous neuropathic pain with amitriptyline treatment supported by the reversal of thermal hyperalgesia in a rodent spinal nerve ligation and subsequent back translation reversing static mechanical allodynia in a rodent STZ type-1 diabetes model. Amitriptyline has since been the most prescribed of the tricyclic agents for diabetic neuropathic pain for the past two decades. The gabapentinoid medicines, gabapentin and pregabalin are calcium channel α2δ-1 and α2δ-2 subunit ligands that were first approved in Europe in 2002 and 2004, respectively. Preclinical efficacy of gabapentin in rodent models of mono-neuropathic pain supported the subsequent clinical observations (Table 1). Following this, preclinical studies demonstrated a superior preclinical profile reversing static and dynamic (light moving stimuli) mechanical allodynia in a rodent STZ type-1 diabetes model, compared with amitriptyline (Table 1). Furthermore, intrathecal administration of gabapentin was 10-fold more potent against dynamic vs. static mechanical allodynia. Given that dynamic allodynia is the most troublesome evoked sign in subgroups of neuropathic pain patients it was considered to be an important and differentiating stimulus evoked sensory endpoint in preclinical poly-neuropathic pain models. However, the fact that gabapentin and amitriptyline have equivalent clinical efficacy in reducing diabetic neuropathic pain (Morello et al., 1999) indicates this superior gabapentin preclinical efficacy does not translate in the clinic. Another aspect where back translation is not straightforward is the delayed-onset analgesia observed clinically, for example a titration phase of 1–2 weeks is required with gabapentin, while acute gabapentin analgesia is typically observed in rodents (Backonja, 1998; Field et al., 1999). Whiteside’s group compared drug exposure in humans with exposure in a rat spinal nerve ligation model demonstrating this mono-neuropathic model, despite lacking face validity (patients with mono-neuropathies represent only 9% of trials (Finnerup et al., 2005)), back translates and predicts efficacious exposure in humans for gabapentin and duloxetine (Whiteside et al., 2008). The proven forward and back translation of these clinically effective drugs demonstrated confidence in the validity of induced animal models of neuropathic pain, similarity between rat and human pain biology and relevance of stimulus evoked sensory measures providing clinically relevant data for diverse mechanisms.

The gold standard treatment pregabalin, is sometimes portrayed as an exemplar forward translational success story for the use of animal models in the neuropathic pain field. As seen in Table 1, rodent STZ type-1 diabetic models were employed in the preclinical development of pregabalin, providing key decision-making allodynia efficacy data for subsequent clinical trials. Pregabalin requires lower doses preclinically and clinically, in contrast to gabapentin (Table 1), as it has a linear, dose proportional absorption in the therapeutic dose range (Freeman et al., 2008). Of note, both drugs require no titration (single dose) preclinically. However, an area of potential bias is that the findings from these preclinical studies only started to appear (Field et al., 1999) at approximately the same time as the pivotal clinical trials of gabapentin (Backonja, 1998; Morello et al., 1999). Irrespective of whether pregabalin is viewed as an exemplar of forward and/or back translation, it is undoubtedly a success story that reinforced the clinical precedence of a novel target α2δ, much needed by the neuropathic pain field. What makes this story even more intriguing is the discovery of the novel mechanism (α2δ-1 and α2δ-2) only one year earlier (Gee et al., 1996) and the subsequent discovery that the analgesic efficacy of pregabalin and gabapentin is mediated by the α2δ-1 sub-unit of voltage gated calcium channels (Field et al., 2006). Mirogabalin, is a potent and selective α2δ-1 ligand with a wider safety margin and superior long lasting efficacy reversing static mechanical allodynia in a rat STZ type-1 diabetes model, compared with pregabalin (Domon et al., 2018). Mirogabalin has also shown promising results on reducing daily pain scores and sleep interference in RCTs for the treatment of diabetic peripheral neuropathic pain (Vinik et al., 2014; Merante et al., 2017; Baba et al., 2019) indicating that a more selective approach may well offer patients a safer and more efficacious option in the future.

The chemical ingredient in chilli pepper, capsaicin, has been available since the 1980s in various formulations as lotions, creams or patches in low concentrations of 0.025–0.075% over the counter to treat neuropathic pain, such as diabetic neuropathy. Clinical efficacy therefore was recognized long before identification of its molecular target in 1997 (Caterina et al., 1997). Capsaicin selectively and potently activates the transient receptor potential cation channel subfamily V member 1 (TRPV1) ligand gated channels on nociceptive fibers leading to TRPV1 desensitization (Caterina et al., 1997). Derry and Moore (2012) concluded that low-concentration topical capsaicin had no clinical efficacy beyond that of placebo but a single application of a prescription strength high concentration capsaicin patch (Qutenza, 8%) is clinically effective in postherpetic neuralgia and diabetic painful neuropathies (Burness and McCormack, 2016; Vinik et al., 2016) with additional QoL improvements (Derry et al., 2017). However, compliance can be low due to the erythema and burning sensation experienced on topical application.

### Forward Translation (Bench to Bedside) of Neuropathic Pain (Figure 1)

**FIGURE 1:**
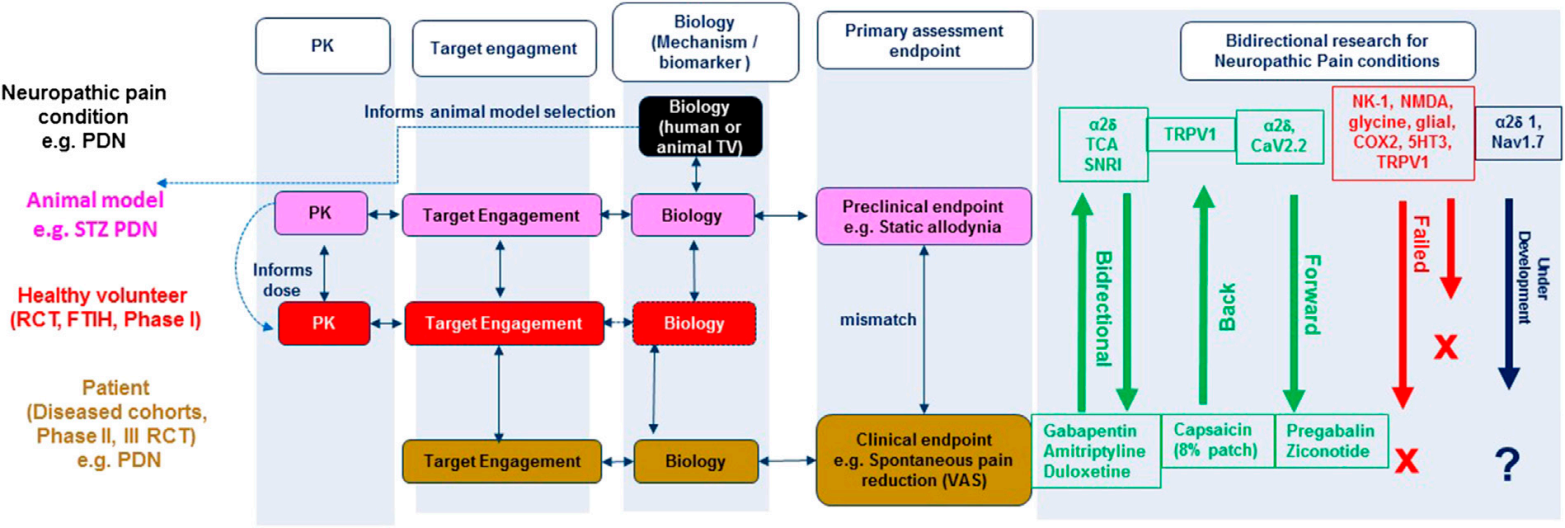
Integrated bidirectional research approach for neuropathic pain: Linking animal and human biology data. RCT (randomised clinical trial), FTIH (First time in human), TV (target validation), STZ (Streptozocin), PDN (painful diabetic neuropathy), PK (Pharmacokinetic), VAS (Visual analogue scale), TCA (tricyclic antidepressant), SNRI (serotoninnorepinephrine reuptake inhibitor). Bidirectional research illustrates the targets/mechanisms that have successfully translated forward (from preclinical research), back (from clinical research), under development (e.g. α2δ1, blue) and failed (e.g., Neurokinin 1 (NK-1), red) novel targets/mechanisms identified from preclinical research.

For the past two decades rational drug discovery efforts have been mechanistically driven addressing targets arising from a better understanding of the mechanism of existing analgesic drugs e.g. TRPV1 (capsaicin patch), α2δ-1 (gabapentinoids) or novel mechanisms arising from biological, human pathophysiological, or genomic studies e.g. Nav1.7, Neurokinin 1 (NK-1) (Figure 1). Despite common neuropathic pain symptoms patients are generally recruited for trials on disease stratification (Finnerup et al., 2015). Similarly, disease or mechanism based animal models that more closely recapitulate the human neuropathic pain clinical condition (face, construct and predictive) are preferred for bidirectional translation. For example, in the last two decades diabetes research has focused on glucose and the STZ type-1 diabetic rat as the preclinical model to understand diabetes disease pathogenesis including painful peripheral poly-neuropathy (Lenzen et al., 2008; Table 1; Figure 1), despite evidence that different mechanisms underly type-1 and type-2 forms (Callaghan et al., 2012).

Figure 1 illustrates the integrated bidirectional research approach of animal and human biology for rational target/mechanism identification, drug discovery and development for neuropathic pain conditions. Knowledge related to the anatomy, physiology, pharmacology, molecular biology, and genetics of pain conditions in experimental animals (typically rodents) or humans (e.g. erythromelalgia) informs pain model selection for target validation, engagement and evaluation of efficacy using clinically relevant endpoints. Bidirectional research in Figure 1 illustrates the target mechanisms that have successfully translated both forward (from preclinical research) and back (from clinical research).

There have been few forward translation successes (see Figure 1). Ziconotide (Prialt, licenced in 2005) is the synthetic form of an ω-conotoxin peptide derived from *Conus magus*, a cone snail SNX-111 that blocks the N-type (CaV2.2) neuronal voltage gated calcium channel. Ziconotide produced striking analgesia in reflexive pain animal models and RCTs for cancer and AIDS related neuropathic pain when administered intrathecally (Malmberg and Yaksh, 1995; Staats et al., 2004). Despite clinical precedence for this target there has unfortunately been limited progress in the development of selective small molecule orally bioavailable N-type calcium channel blockers (Jurkovicova-Tarabova and Lacinova, 2019).

**FIGURE 2:**
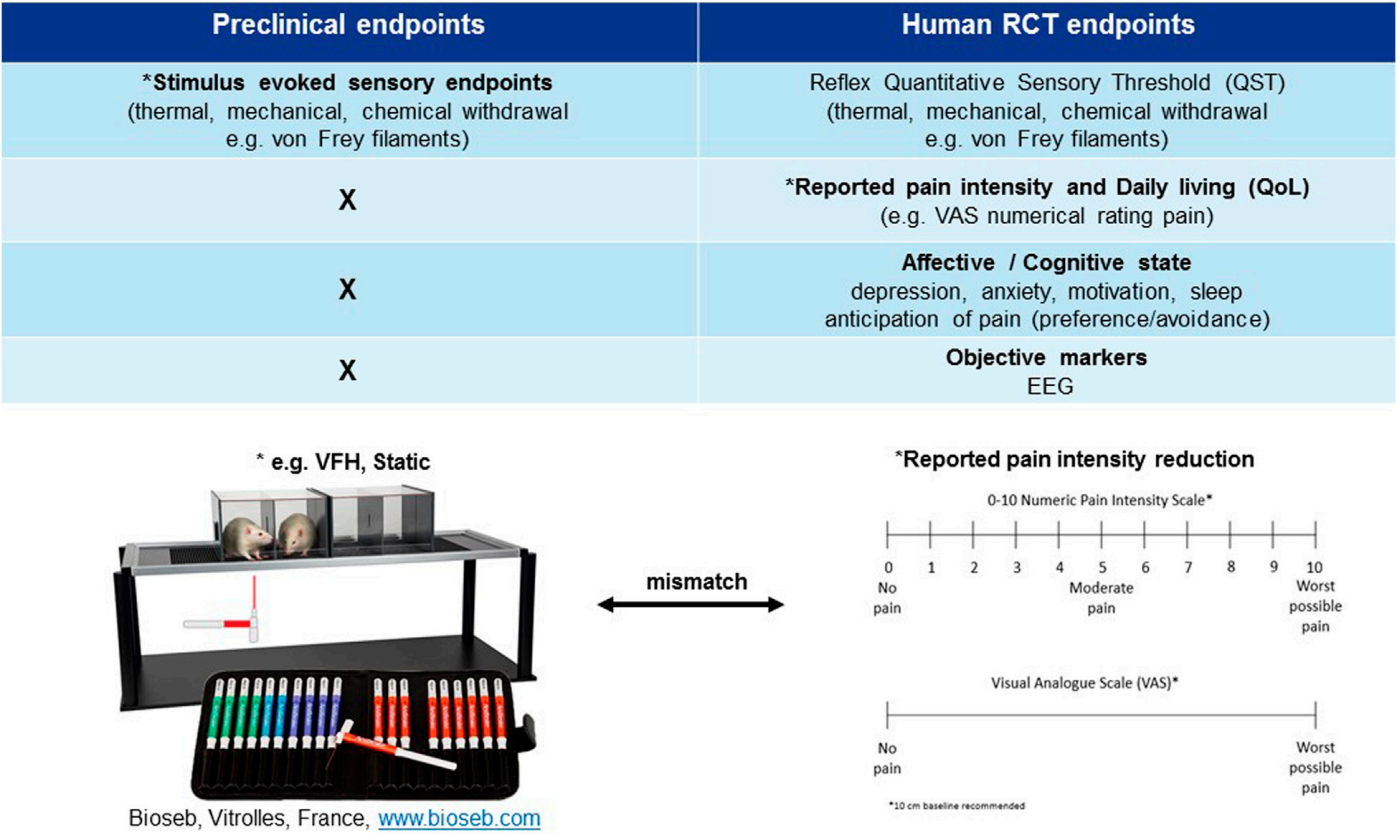
Mismatch of primary neuropathic pain assessment endpoints in preclinical vs. clinical trials. *Primary endpoint, RCT (randomised clinical trial), EEG (electroencephalography), QoL (quality of life), VFH (von Frey hair), Static (static mechanical allodynia).

There is potential for translation of novel neuropathic pain mechanisms from human biology target validation (Figure 2, e.g., Nav1.7) although the number of mechanisms validated in this way is likely to be extremely limited. The sodium channel (Nav1.7) is a neuropathic pain molecular mechanism with conceivable success because of the rare mutations in the Nav1.7 channel identified in patients with inherited erythromelalgia (Nassar et al., 2004; Cox et al., 2006; Minett et al., 2012; Geha et al., 2016), a subset of idiopathic small fiber neuropathy patients and is supported by target validation in genetic animal models of neuropathic pain (Grubinska et al., 2019). A state dependent non-selective Nav inhibitor, Raxatrigine (a.k.a. CNV1014802, BIB074, Deuis et al., 2016) has forward translated in a phase II clinical trial of patients with painful lumbosacral radiculopathy demonstrating that it is well tolerated and produces a remarkable reduction in pain compared to placebo (Versavel, 2015). Furthermore Nav1.7 gain of function mutations give rise to a diabetes induced increased sensitivity of dorsal root ganglion neurons (Hoeijmakers et al., 2014), more severe burning pain and greater sensitivity to pressure stimuli during QST (Blesneac et al., 2018). Hence, targeting this channel may offer hope for reducing pain severity in these different patient groups. However, since Biogen, Xenon and Pfizer have all discontinued research on this target after failure in phase II clinical trials it would be wise to cautiously view Nav1.7 as a panacea to pain (McDonnell et al., 2018).

Despite rational drug discovery efforts identifying numerous novel mechanisms that are efficacious and tolerable in industry standard preclinical models of neuropathic pain, these have all subsequently failed in the clinic (Figure 1, NK-1 antagonists, NMDA antagonists, Glycine antagonists, Glial-modulators, COX-2 inhibitor, 5HT3 antagonist, TRPV1 antagonist, NMDA antagonist, cannabinoid agonist, for recent review see (Yezierski and Hansson, 2018)). Many of these target mechanisms failed in the clinic due to dose limiting toxicity (e.g., cannabinoid agonist, NMDA antagonist, TRPV1 antagonist) and hence should not necessarily be permanently abandoned, for example TRPV1. The clinical development of the highly selective TRPV1 antagonist, AMG-517 was halted by hyperthermic responses in healthy volunteers, believed to be modulated by a peripheral on target mechanism of action (Gavva, 2009). However, recent reports (DelloStritto et al., 2016) indicate that TRPV1 function is rapidly downregulated peripherally in diabetes and our findings have shown the hyperthermic side effects of the TRPV1 antagonist, ABT-102 are absent at an analgesic dose in a rat STZ type-1 diabetes neuropathic pain model (Pritchard et al., 2016). The next step will be to demonstrate this concept in a type-2 animal model of diabetes, opening the potential for the safe use of a TRPV1 antagonist to treat neuropathic pain in the wider diabetic population. Given the lack of forward translation of numerous targets (Pop-Busui et al., 2017) and the established clinical effectiveness of capsaicin patch for painful diabetic neuropathy (back translation, Figure 1), this cannot come soon enough.

In contrast other novel mechanisms, such as substance P (NK-1) antagonists have failed in the clinic despite convincing evidence of analgesic efficacy in animal models. NK-1 receptor antagonists, such as the antiemetic aprepitant at clinically safe and tolerable doses, occupying and engaging >90% of its central target are inefficacious in relieving ongoing pain in postherpetic neuralgia patients despite convincing analgesic efficacy in animals (Rice and Hill, 2006). The lack of forward translation from animal to patients may be explained by species differences in 1) the pathophysiology of substance P and 2) pain measurements of clinical spontaneous pain reduction vs. stimulus evoked sensory endpoints studied preclinically. Differences in the distribution and expression of NK-1 receptors between species is observed at supraspinal sites (pain perception and conscious sensation) and not the dorsal horn of the spinal cord (nociception) (Hill, 2000). An upregulation of supraspinal NK-1 receptors in certain disease states e.g. neuropathic pain, may imply that an even higher receptor occupancy (>90%) is needed for clinical efficacy compared with emesis. Further, clinical spontaneous pain is a conscious sensation that relies on supraspinal cortical processing (Borsook and Becerra, 2006) whilst preclinical stimulus evoked sensory withdrawal reflects a spinal cord reflex activation (Lascelles and Flecknell, 2010). This may imply that the site of pain perception from preclinical to clinical has been inadequately assessed for this mechanism. It is essential to consider whether this mismatch between preclinical and clinical primary pain assessment endpoints is a key contributor to the high number of false positives and lack of forward translation across mechanisms (Figure 2) (see next section). This points to a need to improve bidirectional research i.e. “bedside to bench” to further the clinical relevance of pain assessment in animals and improve “bench to bedside” forward translation.

## Industry Standard Measurements of Neuropathic Pain for the Development of Analgesic Treatments (Figure 2)

### Randomised Clinical Trials (RCTs)

Patients experiencing chronic neuropathic pain are frequently troubled by more than just their pain; comorbid conditions commonly accompany or are caused by the pain. Most notable are sleep disruption and depression/anxiety therefore, diagnosis relies on the patient’s subjective rating of the unpleasant emotional *and* physical sensations (Figure 2). The predominant clinical feature of most neuropathic pain conditions is spontaneous pain (either continuous and/or paroxysmal) as opposed to evoked pain, hence RCTs focus on endpoints relating to verbal self-reporting (visual analogue scales (VAS)) of spontaneous pain intensity reduction (Figure 2; Table 1). Visual analogue or numerical pain scales used in human subjects are a self-reporting system (Edwards and Fillingim, 2007) that is individual, typically influenced by comorbidities and cannot be objectively verified (Farrar, 2010). Sensory gain (mechanical/thermal hyperalgesia and allodynia) is much less frequently reported in neuropathic pain conditions; ranging from 24–33% and is rarer than loss of function symptoms like numbness (42%) (Baron et al., 2017). Hence, reflexive endpoints are infrequently measured in RCTs (Baron et al., 2017), although several recent studies have profiled patients based on individual sensory characteristics (Rice et al., 2018). Self-reporting by its nature poses an inherent subjectivity. Therefore, within the clinical setting there is also a clear need to improve trial outcome measures. Patient stratification, based on sensory profiling and subgrouping, has been one such proposal (Baron et al., 2017) which seems reasonable based on the appearance of sensory gain vs. loss of function symptoms in neuropathic pain conditions. Since individual profiling of patients is labor intensive the ideal scenario would be an objective biomarker of neuropathic pain rather than a subjective reflexive endpoint.

### Industry Standard Stimulus Evoked Sensory Endpoints Primary Pain Endpoints in Preclinical Research

The industry standard primary measure of pain preclinically is standardized evoking (heat, cold, or mechanical) stimuli delivered by an experimenter to assess loss and gain of function of different afferent fiber classes (Aβ, Aδ, and C fibers) (Backonja et al., 2013) (Figure 2). Primarily analgesic efficacy is based upon the significant reduction of hyper-sensory phenomena (e.g., allodynia, hyperalgesia) whilst sensory loss (e.g., deafferentation, anesthesia dolorosa) rarely so (Rice et al., 2008). This is opposite to the clinical scenario where hypo-sensory usually dominate over hyper-sensory symptoms (e.g., painful diabetic neuropathy/radiculopathy) (Baron et al., 2017). Moreover, the hyper-sensory “pain” responder rate is greater than 70% in preclinical models e.g. STZ type-1 diabetes model (static mechanical allodynia) (Field et al., 1999; Fisher et al., 2015) in contrast to only 20–30% in patients (Baron et al., 2017). Of note, Field et al. (1999) demonstrated the prevalence of dynamic mechanical allodynia (60%) was more in line with the debilitating clinical complaint and differentiated gabapentinoid efficacy over amitriptyline which may be a more clinically relevant reflexive endpoint, although it is rarely measured preclinically or clinically.

Stimulus evoked sensory endpoints are robust, reproducible, high throughput measures, with face (although not in terms of prevalence) and predictive validity (back translation, Table 1). However, chronic neuropathic pain is a system based subjective experience that relies on cortical activation and motivational-affective aspects (Melzack and Casey, 1968; Borsook and Becerra, 2006), which is far more complex than a reflex in response to sensory stimulation (spinal or spinal–brainstem–spinal pathways). Restricting preclinical evaluation of pain and analgesia to exclusively subjective, reflexive outcomes is both oversimplified and can be argued not clinically relevant given the continuous lack of forward translation across diverse mechanisms (Figure 1, lack of face and predictive validity). Despite the unsustainable high forward translation attrition rate stimulus evoked sensory endpoints remain the primary decision-making method of neuropathic pain assessment in animals and highlights the necessity to address this clear mismatch of methodological assessment of neuropathic pain in animals and humans.

## The Mismatch Problem

Preclinical scientists and clinicians have become acutely aware of these methodological mismatch issues and consequently it is becoming more conventional to consider markers in patients that can help design more clinically relevant pain assessment tests in animals (bidirectional research). Since the presence or absence of analgesia in animals can only be inferred from observations made by humans (surrogate behaviors, objective measurements) and cannot be self-reported (Percie du Sert and Rice, 2014) this presents a challenge. Many research groups are now looking to improve the markers used preclinically and clinically with the goal of producing markers that can successfully forward translate preclinical candidates to human clinical trials.

## Alternative Neuropathic Pain Assessment Endpoints to Improve Translatability Between Animals and Humans (Bidirectional Research) (Figure 3)

**FIGURE 3:**
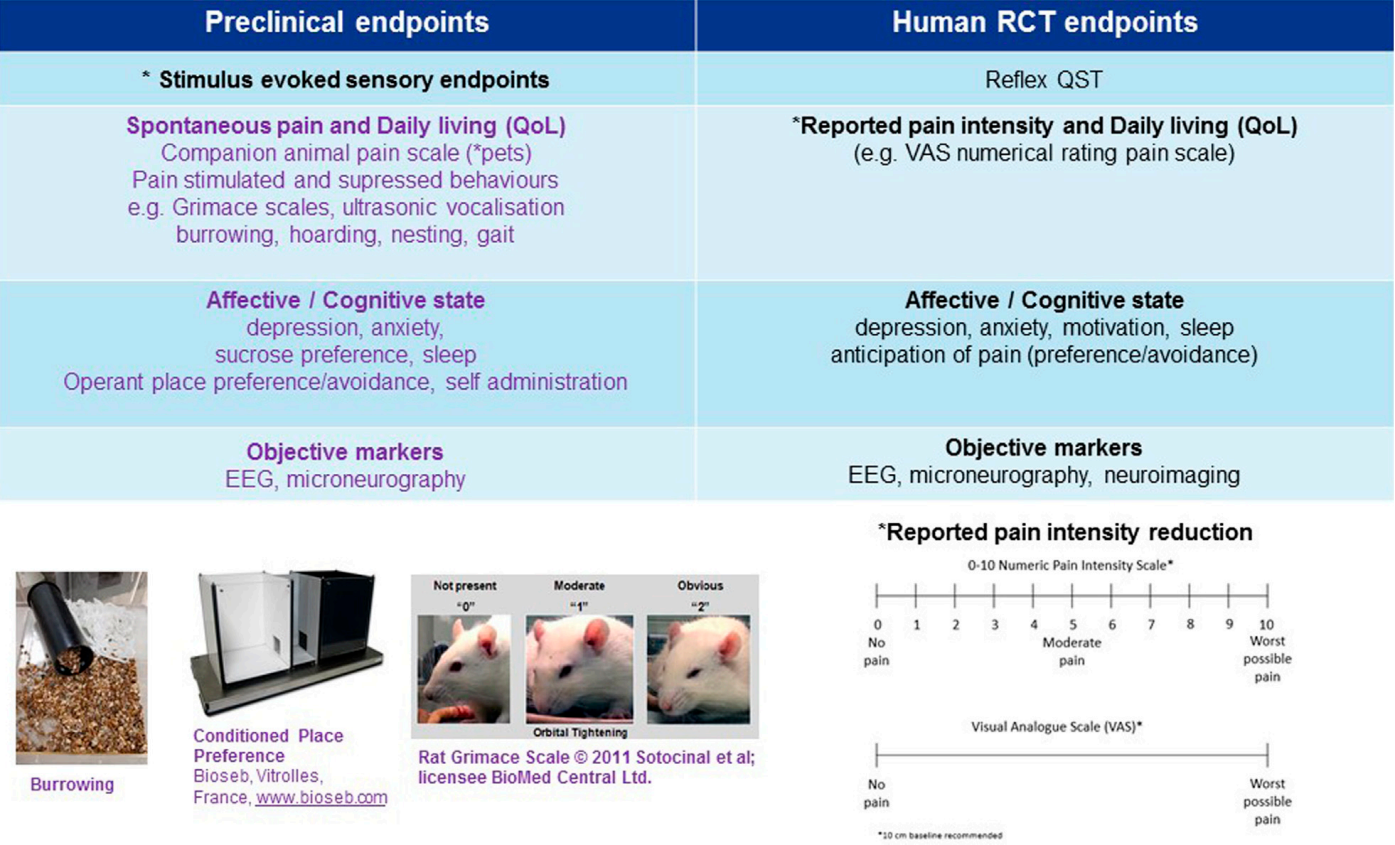
Multiple preclinical neuropathic pain assessment endpoints in development to improve translatability of preclinical to clinical research. *Primary endpoint, RCT (randomised clinical trial), EEG (electroencephalogram), QoL (quality of life).

Alternative preclinical assessments can be used to infer signs of not just the classical reflex hypersensitivity but also spontaneous pain measurements along with the other comorbidities such as anxiety, depression, sleep issues and cognitive deficits so often found associated with chronic pain patients (Figure 3). Here we examine evidence from preclinical and clinical studies focusing on how bidirectional research is addressing current research gaps and helping to develop greater translational, predictive, and more clinically relevant neuropathic pain assessment endpoints. We critique the validity of these alternative behavioral endpoints of spontaneous neuropathic pain and associated comorbidities (QoL, affective and cognitive measures) and how translational techniques, such as EEG, present potential developments in the field for measuring objective signatures of neuropathic pain.

### Spontaneous Pain and Daily Living (QoL): Assessment Questionnaires (Patient and Companion Animals) Correlation to Efficacy Doses

Because pain is an internal, private experience, self-report remains the gold standard for its measurement in the clinic (Fillingim et al., 2016) and the development of easy to use questionnaires, based mainly on self-report of symptoms, has improved diagnosis and management. Two types of questionnaires (screening and assessment) have been validated, rapidly translated and revalidated in several languages (Attal et al., 2018). A number of pain scales are implemented in the clinic for the assessment of the different components of neuropathic pain (for reviews see (Cruccu et al., 2010; Hjermstad et al., 2011; Fillingim et al., 2016; Attal et al., 2018)).

An excellent example of cross-species use of assessment tools has been the use of the numerical rating scale (NRS) in the assessment of pregabalin efficacy in dogs. In human clinical trials, pregabalin efficacy in peripheral neuropathy has been evaluated with success using patients’ daily pain scores (Jenkins et al., 2012). In the first RCT reporting the efficacy of pregabalin in dogs with neuropathic pain, Sanchis-Mora et al. (2019) used the owners “daily pain assessment” with a NRS as the primary efficacy endpoint and stimulus evoked sensory endpoints as the secondary endpoint. Owners assessed spontaneous vocalisations, phantom scratching episodes and exercise impairment (spontaneous behaviors previously validated using VAS (Plessas et al., 2012)) to score the pain severity daily (from 0 no pain to 10 worse pain). Owners’ daily NRS scores were significantly lower during the pregabalin treatment phase compared to placebo. In this study, the daily owner assessment NRS appeared to be a reliable and reproducible assessment tool. An advantage of daily NRS scoring was that potential bias from assessing isolated timepoints was avoided (Colloca et al., 2016; Sanchis-Mora et al., 2019) and the endpoint is translatable across species. However, the disadvantage remains that the assessment of pain being owner dependent is anthropomorphic, therefore inferred by humans and consequently not objective.

Whilst subjective measures of pain in humans does not present the same challenges as subjective measures in preclinical species there is still room for improvement. Subjective measures in humans still introduce variability and limit the translational potential of markers used preclinically to clinical trials, through using different endpoints. Whilst an important improvement to forward translatability would be to use objective preclinical endpoints with the current subjective clinical ones. Using the same objective endpoint in both is the ideal goal for markers of neuropathic pain.

### Pain Supressed Behavioral States: Hoarding, Grooming, Rearing, Nesting, Wheel Running, Burrowing, Gait

Rodents possess several naturalistic/innate characteristics within their behavioral repertoire and suppression of these characteristics can be observed in a similar way to the assessment of QoL measures in neuropathic pain patients. These include wheel running (Stevenson et al., 2011; Pitzer et al., 2016; Green-Fulgham et al., 2020), nesting (Jirkof, 2014), rearing/climbing/exploration (Piel et al., 2014; Pitzer et al., 2016; Deuis et al., 2017) and burrowing (Andrews et al., 2011; Huang et al., 2013; Fisher et al., 2015; Rutten et al., 2018; Sliepen et al., 2019). However, others have found no change in locomotion or rearing in the chronic constrictive injury (CCI) and spared nerve injury (SNI) mice models (Mogil et al., 2010) and that CCI and SNI mice had no change in markers of QoL (Urban et al., 2011). This may be due to nature of rodents as prey animals that will avoid displaying injury or pain to predators (Roughan and Flecknell, 2001).

Analysis of burrowing behaviors in rodent pain models has become increasingly popular over the last 10 years (Pubmed search on pain and burrowing revealed 5 publications in 2010 and 25 in 2019) and demonstrates reproducible results across laboratories (Wodarski et al., 2016). Rodent burrowing is reduced in mono-neuropathic pain states and reversed by clinical analgesics (gabapentin, pregabalin) at 10-fold lower doses (in line with therapeutic exposure in humans) than are needed to decrease hypersensitivity (Andrews et al., 2011; Lau et al., 2013; Rutten et al., 2018). This suggests its usefulness as an objective measure of pain with improved face and predictive validity over stimulus evoked measures of hypersensitivity. Experimental design should always include assessment of drug treatment on burrowing in naïve animals to ensure there is no false positive direct drug effect(s) on this natural behavior. One confound that occurs with all pain supressed spontaneous rodent behavioral tests is that any stimulus that disrupts wellbeing, including disruption of memory (Deacon et al., 2002; Deacon, 2009) can decrease rodent behaviors such as nesting and burrowing. For example, it has recently been demonstrated that a high dose of STZ (75 mg/kg) induces diabetic poly-neuropathic pain, hypersensitivity and abolishes burrowing. However, in contrast to mono-neuropathic pain models burrowing is not reversed by pregabalin, which the authors suggest reflects diabetes associated alteration of the animals’ welfare; and not spontaneous pain (Rutten et al., 2018). Previously, our group have reported a similar decline in burrowing following a high dose (65 mg/kg) of STZ that is resistant to pregabalin but can be reversed by social pairing, indicating pair housing of diabetic rats can improve their welfare and consequently burrowing behavior (Fisher et al., 2015).

This emotional contagion whereby burrowing behavior of STZ type-1 diabetic rats increased if they burrowed with their cage partner (rather than alone) and their individual burrowing increased if they were socially housed with a control partner not in pain (compared with an STZ partner, Fisher et al., 2015) has also been observed by other groups (Langford et al., 2006). The experiments in the laboratory of Mogil et al., demonstrated that mice display higher levels of pain behavior when tested alongside familiar (but not stranger) conspecifics, and featured synchronization of both level and timing of that behavior within the partnership (Langford et al., 2006). Therefore, when evaluating pain suppressed spontaneous behaviors, consideration of the use of appropriate controls is essential e.g. dose of STZ, consistent housing (pair housing at least) during pain testing (stimulus evoked sensory endpoints, burrowing) and testing with control groups is recommended to maximize the welfare and wellbeing of rodents.

As with many other pain supressed behaviors, gait analysis is applicable across a wide range of species and the animal literature is supported by many human studies examining the effect of neuropathic pain on gait (Lalli et al., 2013; Karmakar et al., 2014; Alam et al., 2017). The use of gait analysis to assess pain possesses several advantages most notably the measurements are performed in freely moving animals. Furthermore, when considering development of potential treatments in early drug discovery, only drugs that improve movement will pass screening. Compounds that produce sedative or motor impairments will not restore normal walking and therefore will fail due to side effect profiling. Furthermore, data from gait analysis studies tend to be reproducible across trials and animals within a specific condition (Tappe-Theodor et al., 2019). When considering the use of gait analysis in neuropathic pain models it is worthwhile to note that neuropathic conditions cause both pain and motor effects, either of which can alter gait. The lack of a correlation between the time course for neuropathy induced mechanical hypersensitivity and gait change, in combination with the lack of recovery of normal gait following treatment with standard analgesics would indicate a motor problem (Mogil et al., 2010; Lau et al., 2013; Shepherd and Mohapatra, 2018) which may confound results.

Pain suppressed spontaneous natural behaviors have some important limitations and can be affected by various factors such as caging and animal welfare. However, their aim to provide objective measures of neuropathic pain preclinically is important. If objective pain suppressed behaviors such as burrowing/gait behavior can be successfully refined and developed into robust high throughput markers (initially alongside mechanical hypersensitivity markers), these may improve the translatability potential of preclinical data.

### Pain Stimulated Behavioral States

#### Facial Pain Scoring/Grimace Scale Assessment “Pain Face”

Changes in facial features of humans convey a wide range of states and emotions which have been extensively studied in the context of pain, particularly in neonates (Grunau and Craig, 1987; Maxwell et al., 2013; Chow et al., 2016; Jones et al., 2018; Kappesser et al., 2019) and non-verbal patients (Chow et al., 2016). The idea that facial expressions can reflect the affective (“emotional”) component of the pain experience has been extrapolated to identify pain specific features (“pain face”) using pain scales (“grimace-scales”) for a number of non-human species including rodents (Table 2). The rodent grimace scale originally developed in the laboratory of Jeffrey Mogil by Langford et al., 2010 relies on photo analysis for scoring and consequently is a subjective non-evoked endpoint. Frame grabbing can be done manually, which is very time consuming, however with the development of the Rodent face finder software™ it can now be done automatically (Sotocinal et al., 2011). The system works by recognizing frames containing an eye or ear and tracts pixel movement to ensure extracted images are free from motion blur, thus improving accuracy.

**TABLE 2:** Species in which pain assays producing a grimace scale have been used.

Species	References
Human	Ashraf et al. (2009), Lucey et al. (2011), Bartlett et al. (2014)
Rodent (rats and mice)	Langford et al. (2010), Sotocinal et al. (2011), Matsumiya et al. (2012), Leach et al. (2012), Oliver et al. (2014), Faller et al. (2015), Miller et al. (2016), De Rantere et al. (2016), Akintola et al. (2017), Philips et al. (2017), Schneider et al. (2017), Sperry et al. (2018), Dalla Costa et al. (2018), Cho et al. (2019), Klune et al. (2019), Leung et al. (2019), Ernst et al. (2020)
Pigs/Piglets	Di Giminiani et al. (2016), Viscardi et al. (2017)
Cat	Evangelista et al. (2019), Evangelista et al. (2020)
Sheep	Häger et al. (2017)
Rabbit	Banchi et al. (2020)
Ferrets	Reijgwart et al. (2017)
Horse	Dalla Costa et al. (2014), Dalla Costa et al. (2018)

For review see (Tappe-Theodor and Kuner, 2014; Deuis et al., 2017; Mogil, 2019b; Tappe-Theodor et al., 2019; Turner et al., 2019).

Despite the extensive amount of literature (Table 2), the use of the grimace scale in rodent neuropathic conditions has been hampered by an apparent disparity between pain duration, expression of a painful grimace and mechanical sensitivity (Sotocinal et al., 2011; De Rantere et al., 2016). It is likely that the mismatch arises because of the difference in time course between evoked and spontaneous pain. Furthermore, a lack of grimacing does not mean that the animal is pain free, just that the pain no longer elicits a grimace (Tappe-Theodor and Kuner, 2014). As with ultrasonic vocalisation (USV) (described below) the selectivity of facial grimacing has also been called into question with changes in facial musculature observed in aggressive and fearful contexts (Defensor et al., 2012) and nausea (Yamamoto et al., 2017). Despite this, the use of the grimace scale has shown face validity in several neuropathic pain models including reduction of the grimace scale with fentanyl in the CCI of the infraorbital nerve (Akintola et al., 2017), with meloxicam in a rodent model of radiculopathy (Philips et al., 2017) and Schneider et al. (2017) have shown facial action units are increased following stimulation with acetone in a rat spinal cord injury (SCI) model. Therefore, the rodent grimace scale shows great promise as a valid non-evoked subjective marker of preclinical spontaneous neuropathic pain.

#### Ultrasonic Vocalisation

USV would seem an ideal pain stimulated behavior which can be detected using an inexpensive bat detector (as well as several other commercially available USV detection systems), recorded, and quantified (Sirotin et al., 2014) for review see (Tappe-Theodor and Kuner, 2014; Mogil, 2019b; Turner et al., 2019). The range of rodent sounds fall into the human audible (<20 kHz frequency) and ultrasonic vocalisation (USVs; > 20 kHz frequency) (Roberts, 1975). Many of the studies investigating USVs within the chronic pain space have looked at the “alarm” calls rats emit at approximately 22 kHz. Pain induced USVs have been observed in mice in the SNI model of neuropathic pain (Kurejova et al., 2010) and SCI model of neuropathic pain (Ko et al., 2018) where morphine reduced the SCI induced USVs. Kurejova et al. (2010) used an improved method which enabled the temporal recording of USVs in freely moving mice repeatedly over several weeks and demonstrated a reduction in the SNI induced USVs with gabapentin. The strength of their study, and perhaps the reason why USV effects were observed where others have not seen any (Wallace et al., 2005) lies in the reporting of USVs on a longitudinal timescale and the use of higher frequencies of 37 and 50 kHz, avoiding the 22 kHz frequency range so often used. The criticisms of this endpoint pertain to alarm calls as unselective for pain measurements, being initiated by human handling (Brudzynski and Ociepa, 1992) stress/anxiety (Naito et al., 2003), anticipation of negative events (Knutson et al., 2002) and tightly coupled to sniffing behavior (Sirotin et al., 2014). Furthermore, USVs are highly sensitive to background noise requiring further validation of this technique for use in pain measurements.

### Affective/Cognitive State

#### Anxiety, Depression, Sucrose Preference

As already discussed, neuropathic pain in the clinic is characterized by disturbances of both sensory and affective components. These frequently encountered comorbidities which include appetite decrease, depression, anhedonia and disruptions to sleep cycles present behaviors that can influence pain directly within the preclinical setting (Kontinen et al., 1999). Anxiety/depression like behaviors have recently shown utility within the preclinical setting and the study of neuropathic pain (Narita et al., 2006; Yalcin et al., 2011, Yalcin et al., 2014; Alba-Delgado et al., 2013). Several studies have demonstrated the time dependent nature in the symptomatic development of these comorbidities (Seminowicz et al., 2009; Yalcin et al., 2011; Barthas et al., 2015; Sellmeijer et al., 2018), which have been shown to present long after the mechanical hypersensitivity has worn off (Sellmeijer et al., 2018). This highlights the importance of measuring more than one behavioral change whilst taking into consideration the time at which the measures are captured and analgesia is monitored.

Sucrose preference can measure anhedonia, a key feature of depression (Nestler and Hyman, 2010) which is frequently seen in chronic pain patients but rarely measured in behavioral animal pain studies, despite evidence that depression alters the threshold of pain (Ang et al., 2010). Sucrose preference has been used to explore the influence that chronic pain may have on otherwise rewarding behaviors and has been shown repeatedly to be supressed in chronic neuropathic pain models (Wang et al., 2011; Bura et al., 2013; Amorim et al., 2014; Thompson et al., 2018). We (Fisher et al., 2015) have found that STZ type-1 diabetic rats show a dramatic reduction in 2% sucrose preference within 48 h of STZ administration that is maintained for up to 8 days. This is in line with Wang et al. (2011) who also observed a reduced sucrose preference within 2 days following SNI surgery that persists for up to 2 months, but only in those animals that developed mechanical hypersensitivity, indicating that reduced sucrose preference offers an objective anhedonia marker of spontaneous neuropathic pain. In contrast to Wang et al. (2011), we have consistently observed that STZ type-1 diabetic rats switch back to a 90% sucrose preference (not significantly different from control rats) 9 days after STZ administration despite mechanical hypersensitivity allodynia persisting for at least 18 days (Fisher et al., 2015). This indicates mechanical hypersensitivity may not be a key driver of the acute or chronic anhedonia. It is likely that STZ-dosed animals drink significantly less 2% sucrose than control animals, not due to anhedonia, but as a result of their increased water consumption, due to the emerging diabetes hyperglycaemia and polydipsia phenotype (Lenzen et al., 2008). The development of polyphagia, polydipsia, hyperglycaemia and mechanical hypersensitivity all stabilize 8 days after STZ administration at the same time point that the now diabetic animals switch to a normalized sucrose preference, indicating anhedonia is not an endpoint that can be measured using sucrose preference in this STZ type-1 diabetic rat model of poly-neuropathic pain (Fisher et al., 2015). The fact that localized nerve ligation neuropathic pain models demonstrate persistent reduced sucrose preference alongside mechanical hypersensitivity over many weeks (Wang et al., 2011; Bura et al., 2013) indicates anhedonia offers a pharmacodynamic objective marker in mono-neuropathic pain models. A potential confound when measuring sucrose preference involves potential analgesic (or hyperalgesic) effects of the sweeteners themselves (Suri et al., 2010; Shahlaee et al., 2013) and therefore it is essential to correlate any objective changes in sucrose preference alongside changes in other markers of neuropathic pain, such as stimulus evoked sensory endpoints when validating its suitablility for translatability.

#### Operant and Classical Conditioning (Place Avoidance, Place Preference)

Non-evoked ongoing (spontaneous) pain along with the motivational/affective component of spontaneous pain can be assessed using conditioned place avoidance (CPA), conditioned place preference (CPP) (King et al., 2009; Navratilova et al., 2013) and place escape/avoidance paradigm (PEAP) for reviews see (Sufka, 1994; Tzschentke, 2007; Navratilova et al., 2013; Tappe-Theodor and Kuner, 2014; Tappe-Theodor et al., 2019). CPA is induced by pairing a painful experience with a distinct context which subsequently results in avoidance of the same contextual cues (Johansen et al., 2001; Johansen and Fields, 2004), thereby utilizing the protective function of pain to motivate escape and avoid harm. CPP is the opposite of CPA and assumes that pain relief is rewarding. CPP works by pairing a rewarding experience with a distinctive environment resulting in an increase in the time spent in that environment (Navratilova and Porreca, 2014; Navratilova et al., 2015, Navratilova et al., 2016). The most popular protocol for inducing CPP or CPA as described in rats and mice uses two conditioning chambers distinguished by visual, textural and occasionally odor cues (Figure 3). CPA simply occurs by pairing a painful experience with a specific chamber. In PEAP testing an animal with a paw made hypersensitive is placed in a chamber with a dark (normally preferred) side and a bright side, on top of a wire mesh. At regular intervals one paw is mechanically stimulated with a stiff filament, the hypersensitive paw when the animal is on the dark side and the paw with normal sensitivity when the animal is on the bright side. The shift in the fraction of time the animals spends in the normally preferred dark side provides a measure of the aversiveness of the stimulation of the sensitive paw (Usdin and Dimitrov, 2016).

Synonymous with the time reliant changes observed with anxiety/depression-like behaviors the use of CPP has proved useful in demonstrating the temporal nature in the development of spontaneous ongoing neuropathic pain (Agarwal et al., 2018; Gao et al., 2019). Additionally, it has dissociated the spinal and supraspinal effects of gabapentin (Bannister et al., 2017) and the sensory from the affective/motivational and cognitive aspects of chronic pain (Tappe-Theodor and Kuner, 2014; Shiers et al., 2020). The ability to measure both the affective and non-evoked ongoing/spontaneous component of chronic pain is a major advantage of the CPP/CPA and PEAP paradigms. However, they are all relatively labor intensive and require complicated and time-consuming protocols. Another limitation of the technique is that it does not measure pain in real time; the existence of pain in the past must be inferred by the presence of a CPP in the present (Mogil, 2019a). Long term or chronic neuropathic pain states are not suitable for inducing CPA as they persist outside the context and are therefore not specific to the distinctive environment (Tappe-Theodor et al., 2019). Although there are many difficulties associated with CPP/CPA and PEAP they have and continue to play an influential role in understanding the motivational/affective component of chronic neuropathic pain in animals as well as aiming to improve forward translatability by assessing subjective comorbidities as indirect markers of neuropathic pain in animals, as is the case in RCTs.

#### Cognitive State

Chronic neuropathic pain patients can produce poor performance in tasks requiring cognitive flexibility even when taking commonly prescribed analgesics (Ryan et al., 1993; Povedano et al., 2007; Attal et al., 2014). Cognitive malfunction can be translated and measured in rodents (see Usdin and Dimitrov, 2016 for a review). Indeed, many preclinical studies have demonstrated pathological changes in the hippocampus following peripheral nerve injury (SNI or spinal nerve ligation) that may underlie cognitive deficits (Ren et al., 2011; Mutso et al., 2012; Moriarty et al., 2016; Liu et al., 2017) for example increased TNF-α (Ren et al., 2011; Liu et al., 2017) and altered hippocampal synaptic plasticity and neurogenesis (Mutso et al., 2012).

Studies investigating cognitive dysfunction that can occur during chronic neuropathic pain have been able to dissociate the effects of gabapentin from that of the anti-diabetic drug metformin on cognition (Shiers et al., 2018) and the effects of amitriptyline from those of lornoxicam (Hu et al., 2010). In the elegant studies by Shiers et al. (2018) gabapentin demonstrated a worsening effect in the attentional set shifting task (ASST) in the SNI model whereas metformin completely reversed the cognitive impairment, at doses that completely reversed static mechanical allodynic (Shiers et al., 2018). Further studies from this group using the MNK inhibitor tomivosertib (eFT508) have identified MNK-eIF4E as a novel pathway playing a crucial role in the development of spontaneous pain (measured using CPP) and executive functioning using ASST (Shiers et al., 2020). Whereas no effect was observed on the mechanical hypersensitivity, which the authors attribute to the afferent fiber type affected in the neuropathic pain model (Shiers et al., 2020). In a recent review from Shiers and Price they expand on this dissociation by highlighting the fact that pain relief using currently prescribed transient analgesics is insufficient to reverse cognitive impairments and therefore, there is a clear need to investigate treatment options that can target both pain and its prefrontal cortex driven indirect comorbidities (Shiers and Price, 2020) to improve patient outcome measures.

Using the same mono-neuropathic model, Higgins et al. (2015) have demonstrated that following SNI surgery rats show the equivalent behavioral response to sham controls for food reward under a progressive ratio schedule, thereby implying a similar level of motivation. In contrast, a performance deficit was observed in the 5-choice serial reaction time task (5-CSRTT, a test of attention and reaction time) in the SNI animals only. The deficit became apparent in the second month post-surgery, consistent with an attentional deficit (Higgins et al., 2015) again highlighting the importance of temporal profiling in chronic neuropathic pain models measured over long periods of time.

## Techniques With the Potential to Produce Objective Measures of Neuropathic Pain

Currently the most promising advances toward objective measures of neuropathic pain have been made using microneurography, neuroimaging and EEG. These techniques can be conducted on both preclinical species and on humans and thus data from these techniques can be directly translated between species providing a key opportunity for the development of objective markers for neuropathic pain.

### Microneurography

Microneurography (minimally invasive recording of intact peripheral nerve fibers *in vivo*) provides the opportunity to study the pathophysiology of sensory and axonal abnormalities in pain processing; abnormalities which may underlay the phenomenon of spontaneous pain (Jørum and Schmelz, 2006; Cruccu et al., 2010; Serra et al., 2010) and can also be used as a powerful diagnostic tool for use in patients with neuropathic pain (Serra et al., 2004; Jørum and Schmelz, 2006; Cruccu et al., 2010). Microneurography studies have identified spontaneous activity (primarily in C fibers) that is related to pain, suggesting a potential peripheral mechanism for neuropathic pain (Kleggetveit et al., 2012; Serra, 2012). Importantly from a translational perspective, microneurography can also be used in the rat (Serra et al., 2010; Garcia-Perez et al., 2018) and pig (Jones et al., 2018) providing the opportunity for direct translation of *in vivo* evidence of efficacy, from the preclinical through to the clinical setting.

Microneurography can be time consuming and relies on a fully trained technical expert investigator. Furthermore, microneurography is currently performed in only a few centers around the world. For these reasons, it has only been used on very few occasions to study neuropathic pain patients (Cruccu et al., 2010) (only 21 publications over the last 10 years). These few extant studies have observed similar conductance velocities in rodents and humans (Handwerker et al., 1991; Cain et al., 2001). There is no published normative data for healthy subjects, and published reports are unblinded group comparisons only (Cruccu et al., 2010).

### Neuroimaging

The brain neuroimaging technologies, including magnetic resonance imaging (MRI), positron emission tomography (PET), and magnetoencephalography (MEG), have contributed substantially to our understanding of the perception and processing of pain in humans. The use of PET and MRI for assessment of the brain response to pain, in man, particularly neuropathic pain have been reviewed previously (Peyron et al., 2000; Moisset and Bouhassira, 2007; Morton et al., 2016). Their applicability within rodents positions their use as a key translational biomarker in understanding the same processes from the clinic to the pre-clinical situation (Tracey and Mantyh, 2007; Thompson and Bushnell, 2012; Da Silva and Seminowicz, 2019; Tracey et al., 2019). In rodents, MRI is the most commonly used with PET rarely employed (Da Silva and Seminowicz, 2019). MRI has the advantage of providing better spatial and temporal resolution than PET. However, PET allows for the imaging of neurotransmitters and non-neuronal cells, e.g. astrocytes, in addition to functional imaging.

Functional magnetic resonance imaging (fMRI) is often viewed as the gold standard for longitudinal studies because it is non-invasive. This technique typically uses the blood oxygen level dependent (BOLD) methodology to identify changes in hemoglobin oxygenation which indicates alterations in neural metabolism over time. Although fMRI possesses good spatial resolution it provides only an indirect measure of neuronal activity associated with a task or stimulus and is associated with poor temporal resolution. PET is hampered by both poor spatial and temporal resolution but its advantage over fMRI is that via glucose metabolism, using fluodeoxyglucose (FDG)-PET, it is a more direct measurement of neuronal activity than the BOLD signal. Well-known major confounds associated with rodent brain imaging are the methods used to secure the subject during the scan. Unquestionably, anesthetic agents and restraint techniques impact the results obtained from rodent studies and must be taken into consideration in their design and interpretation (Lancelot and Zimmer 2010). Some PET methods do allow for tracer uptake before the animal is anesthetized however, imaging a moving animal is also not without drawbacks (Gold et al., 2018).

PET scanning using translocator protein (TSPO)-binding radioligands (e.g. [11C]PBR28) is a promising option for studies of neuroinflammation (Albrecht et al., 2016; VanElzakker et al., 2019). TSPO is an 18 kDa, five transmembrane domain protein, mainly situated in the outer membrane of mitochondria. TSPO is thought to be involved in a wide array of vital cellular functions, including steroidogenesis, mitochondrial respiration and cellular proliferation (Herrera-Rivero et al., 2015; Albrecht et al., 2016). It has recently become the molecule of choice for most PET imaging studies which are aimed at imaging glial activation and neuroinflammation (Albrecht et al., 2016). therefore it is ideal for imaging neuropathic pain studies where inflammation and mitochondrial activation represent a predominant feature e.g. diabetes induced neuropathy (Fernyhough, 2015) and traumatic neuropathy (Ellis and Bennett, 2013).

Under healthy baseline conditions TSPO is expressed constitutively at low levels by multiple cell types including neurones and glial cells (Cosenza-Nashat et al., 2009). During an inflammatory response, TSPO becomes substantially upregulated predominantly, if not exclusively, in glial cells in many animal models and human disorders (Chen and Guilarte, 2008; Rupprecht et al., 2010; Wei et al., 2013; Sandiego et al., 2015; Liu et al., 2016). For example, TSPO expression in spinal cord dorsal horn is upregulated in a rodent model of spinal nerve ligation and returns to baseline once the neuropathic pain has resolved or is reversed by the TSPO agonist, Ro5-4864; suggesting that the TSPO upregulation might act as a marker of neuropathic pain and its subsequent recovery.

Though, not all studies find a correlation between the rodent and human microglia/macrophage TSPO response to lipopolysaccharide (LPS) inflammation (Owen et al., 2017). Owen et al. demonstrate no change in TSPO expression in primary human microglia/macrophages compared to a 9-fold increase in rodent primary microglia/macrophages following LPS stimulation (Owen et al., 2017). The authors suggest that TSPO expression (hence TSPO PET binding) may reflect changes in microglia density/proliferation rather than cell activation, which appears to be different from rodents. Whilst others have found an increase in TSPO PET binding possibly reflecting microglial activation in humans following intravenous LPS (Sandiego et al., 2015). This highlights a lack of correlation between activation and induction of TSPO expression. Differentiating whether TSPO PET binding reflects microglia proliferation/density and/or activation *in vivo* is a challenge and extrapolation of TSPO biology from rodent to human myeloid cells should be done with caution (Owen et al., 2017).

Despite the innate challenges associated with the various imaging techniques this technology is still commonly used in rodents and considerable progress is continuing to be made in this field. As such, this has led to the identification of a core pattern of nociceptive-evoked events and brain regions activated in human pain imaging studies (somatosensory cortex, cingulate cortex, thalamus) that are also activated in the majority of the rodent studies (Tracey and Mantyh, 2007; Thompson and Bushnell, 2012). Moreover, pharmacological imaging in rodents shows overlapping activation patterns with pain and opiate analgesics, similar to that found in humans. For example, many of the pain related regions in the brain possess mu opioid receptors e.g. the periaqueductal gray, amygdala and thalamus (Zubieta et al., 2001) and imaging studies in both rodents (Shah et al., 2005) and man (Casey et al., 2000) have elucidated the activity of opioids alone and following induction of pain on these specific brain regions. Following opioid administration these studies have demonstrated a clear reduction in the pain-evoked activation of the brain region demonstrating the translational nature of this methodology.

### Electroencephalography (EEG)

EEG, first conducted in humans by Hans Berger in 1924, is the technique used to study the electrical currents produced in the brain (Berger, 1929). It is recorded using electrodes: in humans commonly placed on the scalp (Sazgar and Young, 2019); in rodents usually placed in direct contact with the dura mater (Lundt et al., 2016), to produce an EEG signal. The EEG signal represents synchronized electrical activity from populations of neurons (Binnie and Prior, 1994). EEG signal patterns may be useful as objective and translatable central markers of neuropathic pain as EEG signals can be recorded in both animals and humans without introducing observational bias, that current neuropathic pain stimulus evoked sensory endpoints may create (Bove, 2006; Leiser et al., 2011; Wilson et al., 2014; Sullivan et al., 2015).

Early evidence showed neuropathic pain patients had an increased theta (4–8 Hz) power, decreased alpha (8–12 Hz) power and increased rhythmicity of theta oscillations termed thalamocortical dysrhythmia (Llinas et al., 1999). Evidence from animal models indicated the ventral posterolateral nucleus of the thalamus as the cause of thalamocortical dysrhythmia, which transmits spinal cord signals to the somatosensory cortex (Gerke et al., 2003; Caylor et al., 2019). Thalamocortical dysrhythmia has continued to be a commonly referenced model for the changes in theta oscillations seen in neuropathic pain (LeBlanc et al., 2016b; Vuckovic et al., 2018a; Vanneste et al., 2018). However, in humans Stern et al. (2006) identified that although there were many similarities in EEG signal (such as increase theta power), differences in the extent of this increase in theta power were related to the neuropathic pain cause. Thus, further development of EEG may produce individual patterns of EEG changes for different neuropathic pain causes.

Findings in humans that spinal cord stimulation (SCS) reduces neuropathic pain have now been replicated in rodents through using EEG patterns as a marker. This procedure involves a surgically implanted device applying an electrical stimulation to the spinal cord of patients to alter neuronal activity (Caylor et al., 2019). SCS has been used in humans for many years to treat chronic and neuropathic pain, although the mechanism of action is still being investigated (Sivanesan et al., 2019). Human neuropathic pain patients successfully treated with SCS have shown a reduction in delta (0.5–3 Hz), theta (3–8 Hz) (Sufianov et al., 2014) and high theta (7–9 Hz) power (Schulman et al., 2005). Findings in the CCI rat model show that SCS successfully reversed thermal hyperalgesia and reduced the increased EEG power in the 3–4 Hz range (Koyama et al., 2018b). This provides supportive evidence that the increase in theta power seen in many neuropathic pain studies may be a reliable marker that can be used to screen drugs against. To this end Koyama et al. (2018a) have developed a rodent model of pain that uses increased theta (4–8 Hz) power as a marker. This model demonstrated a reversal of theta power and allodynia after treatment with pregabalin and EMA 401 (an angiotensin II type 2 receptor inhibitor with positive efficacy in a phase II postherpetic neuralgia trial, although clinical development has now been halted due to toxicological side effects) (Rice et al., 2014). However, Moreover, minocycline (glial cell inhibitor with poor results in human clinical trials) failed to reverse these markers (Vanelderen et al., 2015). Significantly, sub- and supra-optimal doses equivalent to human exposure were identified only when using theta power and not allodynia as an endpoint, providing strong evidence for the use of EEG signal patterns as a translational marker of neuropathic pain (Koyama et al., 2018a). This back translation of the effect of pregabalin is an important step in validating this method as a marker of neuropathic pain.

Other than the potential of data generated through EEG recordings as a translatable marker, it also has uses in neuropathic pain treatments that avoid the use of animals, by being applied directly to humans. One such method is neurofeedback modulation (NFB) which uses a real time EEG display, to allow patients to monitor and regulate their brain oscillations (Jensen et al., 2008). After training, patients are given targets such as to increase alpha (9–12 Hz), whilst decreasing theta (4–8 Hz) and high beta (20–30 Hz) oscillations. This method of NFB has been found to significantly reduce pain for some neuropathic pain patients (Hassan et al., 2015). Recently, patients have been able to practice NFB at home when it is most needed. In this study 12/15 patients achieved a statistically significant reduction in pain, with upregulation of alpha (9–12 Hz) oscillations being the most successfully achieved (Vuckovic et al., 2019). Although placebo-controlled testing is difficult in NFB, pre-recorded EEG data has been examined during training session and patients reported no changes in pain scoring (Hassan et al., 2015). This provides additional evidence that modulating EEG oscillations can provide therapeutic benefit.

In SCI, EEG signals have been used to classify (Wydenkeller et al., 2009) and even predict patients that will develop neuropathic pain (Vuckovic et al., 2018b; Vuckovic et al., 2018a). Wydenkeller et al. (2009) found that reduced peak EEG frequency between 6 and 12 Hz was able to classify between neuropathic pain and non-neuropathic pain patients with 84% accuracy. Recently, EEG reactivity to eyes opening (Vuckovic et al., 2018b) and feature classification (Vuckovic et al., 2018a), have been used to predict neuropathic pain development in SCI. In 85% of cases on average these methods predicted non-neuropathic pain patients that would develop neuropathic pain. Interestingly this study found that the oscillations involved in the most accurate predictions included alpha (8–12 Hz) and beta (4–8 Hz). This contributes to the developing theory that theta changes occur progressively with neuropathic pain development and alpha and beta changes are seen before neuropathic pain onset (Vuckovic et al., 2018a). Our research is investigating whether these findings can be translated into an animal model such as the STZ type-1 diabetic rat to open an exciting avenue of research into drugs to slow or even prevent the development of neuropathic pain.

The use of data produced by EEG as a marker of neuropathic pain has solid potential; however, it does come with limitations. One key example is the different methods of EEG electrode placement in humans and rodents (Lundt et al., 2016; Sazgar and Young, 2019). The skull and skin have different conductivities which can alter EEG recordings and affect translatability between species (Drinkenburg et al., 2016; Vorwerk et al., 2019). One way to improve this is using epicranial screws in rodents that are placed into but not through the skull which is closer to the placement of electrodes in humans (LeBlanc et al., 2016a; Koyama et al., 2018a, Koyama et al., 2018b). Additionally, differences between rodent and human EEG signals may occur due to physiological differences in brain size and pathways, or procedural differences in EEG recordings (Leiser et al., 2011; Wilson et al., 2014). For example, coherence in the theta (4–9 Hz) band was increased in human neuropathic pain patients but decreased in rats (Llinas et al., 1999; Sarnthein and Jeanmonod, 2008; Leblanc et al., 2014).

An important consideration when using EEG to investigate neuropathic pain is whether the changes seen in the brain or spinal cord are most important. Whilst it is important to continue work in both areas, the current understanding that pain is a combination of both sensory and emotional aspects leads to the likelihood that EEG recordings focusing on changes in the brain will have a greater ability to analyze the true pain experience. However, the changes seen in the spinal cord may provide a more accessible target as shown by SCS where the effects of neuropathic pain are not clouded by additional factors such as mental state and previous experiences.

EEG is a technique that provides a promising way of producing data that can be used as a biomarker of neuropathic pain as it is both translatable and unbiased. Further development is needed to fully characterize the changes produced by individual neuropathic pain causes and identify specific EEG patterns that translate between animals and humans.

## Conclusion

In this review, we have discussed how bidirectional research is attuning animal models closer to the human condition, by refining neuropathic pain assessment outcome measures to aid translation of preclinical results to the clinic. Alternative behavioral measures outlined in this review provide a means by which preclinical researchers can study not only the development of neuropathic pain but the analgesic response to clinical candidates in a more comprehensive manner. However, the very nature of moving away from simple reflex based measures does mean that behavioral measures may be more easily perturbed by subtle changes relating to environmental events e.g. housing conditions and minor protocol variations that can result in inter-laboratory and inter-group variability. For example, short term social isolation has been shown to suppress burrowing behavior in STZ type-1 diabetic but not control rats (Fisher et al., 2015). Given that observational pain behavioral measures are easily modifiable and subjective we advocate a need for further validation of objective pain markers such as data produced by EEG. Despite automation, objective markers of pain, by their very nature, are longer lasting and more labor intensive than the routinely favored stimulus evoked sensory measures. Although, results from such studies can be translated directly into and back from human studies. The more examples of successful back and forward translation that are documented using multiple neuropathic pain endpoints, the more evidence we will have as to whether these provide superior animal to human predictivity, compared to stimulus evoked sensory endpoints alone, and the more confidence we are likely to have in the success of clinical interventions derived from and/or supported by rational drug discovery.
